# Prevalence and Clinical Characteristics of NEUROD1‐MODY in Chinese Early‐Onset Type 2 Diabetes Mellitus and a Literature Review

**DOI:** 10.1111/1753-0407.70080

**Published:** 2025-03-27

**Authors:** Tianhao Ba, Qian Ren, Siqian Gong, Meng Li, Hong Lian, Xiaoling Cai, Wei Liu, Yingying Luo, Simin Zhang, Rui Zhang, Lingli Zhou, Yu Zhu, Xiuying Zhang, Jing Chen, Jing Wu, Xianghai Zhou, Yufeng Li, Xirui Wang, Fang Wang, Liyong Zhong, Xueyao Han, Linong Ji

**Affiliations:** ^1^ Department of Endocrinology and Metabolism Peking University People's Hospital, Peking University Diabetes Center Beijing China; ^2^ Beijing Pinggu Hospital Beijing China; ^3^ Beijing Airport Hospital Beijing China; ^4^ Beijing Tiantan Hospital Capital Medical University Beijing China

**Keywords:** early‐onset type 2 diabetes mellitus, MODY6, NEUROD1‐MODY

## Abstract

**Background:**

Maturity‐onset diabetes of the young resulting from mutations of the NEUROD1 gene (NEUROD1‐MODY) is a rare form of diabetes and has not been well studied. We aimed to estimate its prevalence in Chinese patients with early‐onset type 2 diabetes mellitus (EOD) and summarize its clinical and genetic characteristics.

**Methods:**

We performed next‐generation sequencing in 679 patients with EOD to screen rare variants in NEUROD1 exons and evaluated the effects of variants using in vitro experiments. All the reported NEUROD1‐MODY cases were reviewed. Patients carrying pathogenic or likely pathogenic variants were diagnosed with NEUROD1‐MODY according to the American College of Medical Genetics and Genomics guidelines.

**Results:**

Four rare variants were identified in 679 patients with EOD, but only P197H decreased the transcriptional activity in in vitro functional assays to an extent comparable to the well‐known mutation causing NEUROD1‐MODY. Its frequency was pretty higher in the European population (0.024) than that in the East Asian population (0.00034) according to the gnomAD database. Twenty‐eight previously reported patients could be confirmed as diagnosed. The patients in Asia had a lower body mass index and a higher rate of ketosis compared with Caucasians, and the mutations present in Asia often occurred in the transactivation domain. Neurological abnormalities were observed in 10.7% of the patients with NEUROD1‐MODY.

**Conclusions:**

NEUROD1‐MODY in Chinese patients with EOD is not common (≤ 0.15%). The P197H might account for MODY in Chinese with a higher penetrance than Caucasian and needs further exploration. The possible differences of phenotypes exist between the two ethnic populations.

## Graphical abstract

We, for the first time, calculated that the prevalence of NEUROD1‐MODY was less than 0.15% in Chinese patients with early‐onset type 2 diabetes mellitus. The p.P197H variant of the NEUROD1 gene significantly decreased the transcriptional activity of the insulin promoter by nearly 50% compared with that of the wild type. This variant might result in MODY in Chinese individuals with a higher penetrance than Caucasians. The possible differences of NEUROD1‐MODY phenotypes exist between Asians and Caucasians.

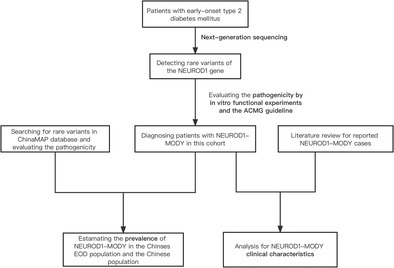

**Summary**

- NEUROD1‐MODY is a rare type of diabetes.
- We, for the first time, calculated its prevalence in Chinese patients with early‐onset type 2 diabetes (≤ 0.15%).
- The p.P197H variant of the NEUROD1 gene significantly decreased the transcriptional activity of the insulin promoter by nearly 50% compared with that of the wild type.
- This variant might result in MODY in Chinese individuals with a higher penetrance than Caucasians.
- The possible differences in NEUROD1‐MODY phenotypes exist between Asians and Caucasians.

## 1 Introduction

Maturity‐onset diabetes of the young (MODY) is a special type of diabetes mellitus (DM) that accounts for 1%–5% of people with diabetes [1, 2]. Further research on rare types of MODY such as NEUROD1‐MODY is needed to improve our understanding of this disease. The neuronal differentiation 1 (NEUROD1) gene is located on chromosome 2q32 and contains two exons and one intron. It is a basic helix–loop–helix (bHLH) transcription factor involved in pancreatic and neural development. The structure of NEUROD1 contains an N‐terminus (amino acid 1–99), a bHLH domain (amino acid 100–155), and a C‐terminus (amino acid 156–355). The latter two domains contain evolutionarily conserved sequences [3], and the Transactivation domain is located at the C‐terminus (amino acid 188–355). NEUROD1 homozygous mutations are known to induce permanent newborn diabetes mellitus (PNDM) [4, 5], whereas heterozygous mutations can result in NEUROD1‐MODY.

In 1999, Malecki et al. first reported two families carrying p.Arg111Leu (R111L) and p.His206Profs*38 heterozygous mutations in NEUROD1 as NEUROD1‐MODY [5]. After that, more cases of NEUROD1‐MODY have been reported. Several studies have screened NEUROD1 gene mutations in patients with diabetes suspected of having MODY but with a relatively small sample size (varying from 25 [6] to 289 [7, 8]). In several recent large‐scale genomic studies of patients with late adolescence/adult‐onset diabetes [9], pediatric populations [10], or early‐onset type 2 diabetes mellitus (EOD) [11], NEUROD1 was not included in the screening gene panel. Consequently, the prevalence of NEUROD1‐MODY in patients with EOD remains unknown, especially in Chinese.

Notably, some previously reported variants were classified as uncertain significance (VUS) rather than pathogenic (P) or likely pathogenic [12] variants according to the American College of Medical Genetics and Genomics (ACMG) [13] guidelines in the patients with NEUROD1‐MODY [13], which might attribute to only a few mutations having undergone in vitro functional studies [5, 14]. Therefore, the diagnoses in these cases were not entirely conclusive. In 2019, a literature review summarized reported NEUROD1‐MODY cases [15]. However, this review did not analyze the pathogenicity of the reported mutations, and some cases might have incorrectly been included. Thus, the clinical phenotype features of NEUROD1‐MODY need precise reevaluating based on correct diagnosis. Moreover, additional cases were reported since 2019, and a new systemic review is necessary.

Therefore, this study aimed to estimate the prevalence of NEUROD1‐MODY in the Chinese EOD population and summarize the genetic and clinical characteristics of NEUROD1‐MODY patients. In vitro functional experiments were used to evaluate the mutations identified in our study cohort, and a strict diagnostic criterion was used to diagnose NEUROD1‐MODY according to the ACMG guideline. We aimed to provide evidence and recommendations for further diagnosis and screening.

## 2 Methods

### 2.1 Study Population

We collected data from 679 unrelated patients with EOD who attended outpatient or inpatient clinics of Peking University People's Hospital between September 2013 and June 2019. All the participants were Chinese and met the 1999 World Health Organization (WHO) diagnostic criteria for DM. The inclusion criteria were as follows: (i) age at diagnosis ≤ 40 years; (ii) neither classical clinical manifestations of type 1 diabetes nor positivity for islet cell antibodies [16], anti‐insulin antibodies (IAA), and glutamic acid decarboxylase antibodies (GADA); and (iii) absence of typical clinical features of other specific forms of diabetes (e.g., chronic pancreatitis). This study was approved by the Ethics Committee of Peking University People's Hospital ([2013] No. 12), and written informed consent was obtained from all participants.

Patients completed a physical examination and questionnaires at the time of inclusion in this study, and their demographic characteristics were collected and described in Table S1 and a previous study [17]. Fasting venous blood samples were collected after an overnight fast of 8 h. Body mass index (BMI) obesity classification criteria for different ethnic groups were according to their respective guidelines [18, 19, 20].

### 2.2 Detection of NEUROD1 Variants in Exons

All DNA samples were extracted from peripheral blood samples. We used Roche NimbleGen human exon V2 capture kit or customized Agilent capture to perform whole‐exome sequencing or target sequencing of samples on the Illumina Hiseq2500 system and Hiseq4000 platform, with data coverage (∼100×) > 99% and depth > 200 bp. Variants with minor allele frequencies (MAF) < 0.001 were selected after screening using the ExAC East Asian (http://exac.broadinstitute.org/), 1000 Genome Project Chinese (https://asia.ensembl.org/), and China Metabolic Analytics Project (ChinaMAP) databases (http://www.mBiobank.com). We applied Polyphen2, CADD, SIFT, PROVEAN, and Mutation Taster Prediction software to screen for at least one software‐predicted deleterious mutation. The pathogenicity of the variants was assessed based on the ACMG guideline [13]. Subsequently, all selected rare NEUROD1 variants were verified by Sanger sequencing (Figure S1). The primers used for Sanger sequencing are listed in Table S2.

### 2.3 The Estimation of Prevalence of NEUROD1‐MODY in General Chinese Population

We searched the ChinaMAP database to identify NEUROD1 variants with MAF < 0.001 and evaluated these mutations according to the ACMG guidelines. The ChinaMAP database (www.mbiobank.com) contained 10 588 deep whole genome sequencing (WGS) data (40.80×). These individuals were randomly selected from eight main ethnic populations across 27 provinces of China, from ~450 000 participants. It is a representative and standard population‐based human genetics database for Chinese. We identified P and LP mutations to determine the prevalence of NEUROD1‐MODY in the unselected large Chinese population.

### 2.4 Functional Study of Rare Variants of NEUROD1

To construct a wild‐type NEUROD1 overexpression plasmid, a full‐length human NEUROD1 transcript was cloned into the pcDNA3.1 expression plasmid. NEUROD1 variant plasmids were created using a point mutation kit and detected by Sanger sequencing. The R111L variant, which was verified as a well‐recognized MODY‐causing mutation by Malecki et al. was chosen as a positive control [5]. A reporter plasmid was created by inserting the human insulin promoter (INS) sequence (NG_007114.1 chr11:2182452–2182809) into the PGL4 plasmid. Wild‐type or NEUROD1 variant expression plasmid (0.5 μg) and INS luciferase reporter plasmid (0.5 μg) per well were co‐transfected using lipo3000 (Thermo Fisher, USA) into HEK293T cells cultured in 12‐well plates and harvested after 48 h of transfection to measure luciferase levels (Promega GloMax, USA). The pRL‐TK Renilla luciferase (RLuc) reporter vector (Promega, Madison, WI, USA) was transfected at a ratio of 1:100 and used as an internal reference. Transfection efficiency was tested by quantitative real‐time polymerase chain reaction (qPCR). The primers used for qPCR are listed in Table S2. The Dual‐Luciferase Reporter Assay System (Promega, USA, E1910) was applied to perform in vitro functional experiments on the identified variants to verify their regulatory effects on human insulin promoter expression. Each experiment was repeated three times, and the results were expressed as the mean.

### 2.5 Literature Review

We searched the PubMed, ClinVar, and China National Knowledge Infrastructure databases for NEUROD1‐MODY (up to October 30, 2023), including research articles, case reports, letters, and reviews. The search terms were MODY6, maturity‐onset diabetes of the young 6, NEUROD1, diabetes, and NEUROD1‐MODY to draw up and compare the clinical characteristics of patients of different races. We also collected information regarding the mutations, including location, amino acid changes, software prediction results, and whether functional experiments were performed for further analysis and pathogenicity assessment.

### 2.6 Statistical Analysis

Continuous variables satisfying a normal distribution were described by mean ± SD, and *t*‐test was used to compare means between two groups. Variables that did not conform to a normal distribution were described as medians and interquartile distances, and the Mann–Whitney *U* test was used to compare differences between two groups. Categorical variables were expressed as numbers (proportions). Differences between groups were measured by the *χ*2 test, the continuity‐corrected chi‐square test, or Fisher's exact test. *p* values (two‐sided) < 0.05 were considered significant. Statistical analyses were performed using IBM SPSS Statistics, version 25.0.

## 3 Result

### 3.1 Screening for NEUROD1 Rare Mutations and Diagnosis of NEUROD1‐MODY Patients in Our EOD Cohort

Four rare NEUROD1 variants were identified in our EOD cohort consisting of 679 patients; among these variants, three were missense mutations (P197H, R158C, and E13K) and one was a non‐frameshifted mutation (K39del) (Table 1). Variants E13K, R158C, and K39del were novel and reported in the present study for the first time. The MAF in each database and results of the prediction software are shown in Table S3. According to the ACMG guidelines, all four variants were preliminarily classified as VUS.

**TABLE 1:** Rare variants of NEUROD1 detected in Chinese early‐onset type 2 diabetes patients.

Proband	P1	P2	P3	P4
Variant	P197H	R158C	E13K	K39del
Position	182542998	182543116	182543551	182543471–182543473
Base change	c.590C>A	c.472C>T	c.37G>A	c.115_c.117delAAG
Protein change	p.P197H	p.R158C	p.E13K	p.K39del
Rs number	rs8192556	rs756392502	rs763871039	NA
ACMG classification^a^	Likely pathogenic^b^ (PS3 + PM2 + PP3)	Uncertain significance (PM2 + PP3)	Uncertain significance (PM2 + PP3)	Uncertain significance (PM2)
MAF (%)				
1000G_Chinese	0	NA	NA	NA
ExAC_East Asian	0.0003	NA	0.000008239	0
ChinaMAP	0.00028 (6/21176)	NA	NA	NA

*Note:* RefSeq: GRCh37. NC_000002.11 (182533022.0.182545244) NM_002500.5 ENST00000295108.4. NA, the variant has no minor allele frequency (MAF) in the database.

^a^ The classification was according to the American College of Medical Genetics and Genomics standards and guidelines.

^b^ The MAF of P197H differs between Caucasian (0.024) and East Asian (0.00034) populations, leading to a controversial conclusion about pathogenicity according to ACMG guideline.

**FIGURE 1:**
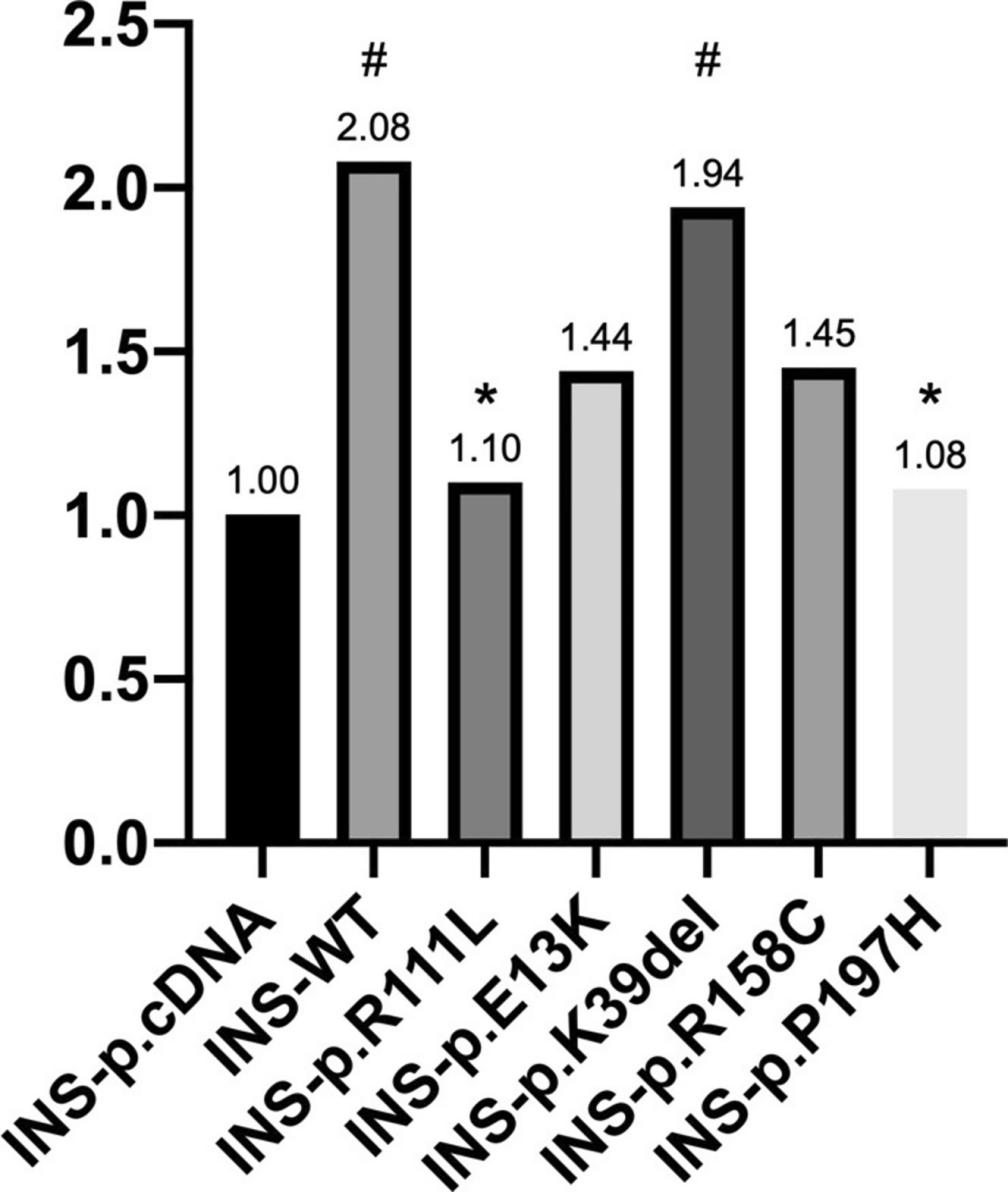
The luciferase activity of NEUROD1 wild type and variants. The Dual‐Luciferase reporter assay showed the activation of the insulin promoter by wild‐type NEUROD1 and variants (R111L, E13K, K39del, R158C, and P197H). The luciferase activity was relative to the INS‐pcDNA3.1. # Significantly different from control. * Significantly different from wild type.

We then validated their effects on the role of NEUROD1 in activating the insulin promoter using a Dual‐Luciferase system. The results demonstrated that P197H significantly decreased the transcriptional activity of the insulin promoter by nearly 50% compared with that of the wild type (1.08 vs. 2.08, *p* = 0.017). This reduction in activity was observed in the positive control, R111Lvariant, similarly (1.08 vs. 1.10, respectively, *p* = 0.898). The K39del did not affect the promoter activity compared to the wild type. Although reductions were observed for the E13K and R158C variants, these differences were not statistically significant (Figure 1).

The clinical characteristics of the four patients carrying rare NEUROD1 mutations are shown in Table 2. The patient with the P197H mutation was female, diagnosed with diabetic ketosis at 26 years of age, and referred to our clinic at 28 years of age. She had no significant medical history or neurological abnormalities. She was born full‐term but had a low birth weight. Her mother and second aunt also had diabetes. Her BMI was 16.2 kg/m^2^. The fasting C‐peptide (FCP) level was 0.82 ng/mL, fasting blood glucose (FBG) was 13.44 mmol/L, and glycated hemoglobin (HbA1c) levels were 13%. Her total cholesterol was 4.44 mmol/L, low‐density lipoprotein cholesterol (LDL‐C) was 0.78 mmol/L, and triglycerides were 0.78 mmol/L. Laboratory tests and examinations revealed no diabetes complications. The patient received multiple insulin injections (MDI).

**TABLE 2:** Clinical characteristics of Chinese early‐onset type 2 diabetes patients with rare variants of NEUROD1.

Proband	P1	P2	P3	P4
Variant	P197H	R158C	E13K	K39del
Sex	F	M	M	M
Age at diagnosis	28	32	31	34
Family history	Yes	Yes	Yes	Yes
BMI (kg/m^2^)	16.2	28.7	28.4	24.2
BP (mmHg)	110/80	125/85	120/82	—
Ketosis^a^	Yes	—	—	—
Antibody	No	No	No	No
HbA1c (%)	13	8.6	9.5	6.2
FBG (mmol/L)	13.44	9.0	13.72	6.89
FINS (μU/ml)	7.54	—	9.17	4.26
FCP (ng/ml)	0.82	2.33	—	1.21
TC (mmol/L)	4.44	4.8	13.03	3.59
LDL‐C (mmol/L)	3.25	3.05	—	
HDL‐C (mmol/L)	0.98	1.01	0.68	1.15
TG (mmol/L)	0.78	2.17	—	—
BUN (μmol/L)	34	4.45	—	5.05
UACR (mg/g)	12.24	0.17	23.66	5.35
eGFR (ml/min × 1.73 m^2^)	147.57	138.61	169.89	—
Treatment	Insulin	OAD	—	

Abbreviations: BMI, body mass index; BP, blood pressure; BUN, blood urea nitrogen; eGFR, estimated glomerular filtration rate; F, female; FBG, fasting blood glucose; FCP, fasting c‐peptide; FINS, fasting serum insulin; HbA1c, hemoglobin A1c; HDL‐c, high‐density lipoprotein cholesterol; LDL‐c, low‐density lipoprotein cholesterol; M, male; OAD, oral antidiabetic drugs; P, patient; TC, total cholesterol; TG, triglycerides; UACR, urinary albumin/creatinine ratio.

^a^ Ketosis presenting at diabetes diagnosis.

Notably, the MAF of P197H among ethnic populations in databases is different, rare in Chinese (0.00028) and East Asian populations (0.00034), and low frequency in the gnomAD full database (0.019) and European (non‐Finnish) populations (0.024). According to the ACMG guideline, the P197H variant was determined to be a VUS based on the MAF in the gnomAD full database and European (non‐Finnish) populations (PS3 + BS1 + PP3), while it was determined to be a likely pathogenic (PS3 + PM2 + PP3) variant in the Chinese or east Asian population. Thus, only one or no patient was diagnosed with NEUROD1‐MODY in this cohort (≤ 1/679, 0.15%).

### 3.2 The Prevalence and Pathogenicity of NEUROD1 Rare Variants in ChinaMAP Database

In total, 406 NEUROD1 variants were detected in the ChinaMAP database. There were 39 rare mutations, excluding synonymous mutations and mutations in noncoding regions, with a MAF < 0.001, carried by 93 people (0.88%), including 38 missense mutations and one frameshift mutation, resulting in protein truncation (Table S4). According to the ACMG criteria, p.S274fs carried by one participant was classified as LP (0.009%). Meanwhile, six participants carried P197H, which is controversial in terms of pathogenicity. Other variants were defined as VUS in 0.81% of subjects (86/21176). Consequently, the probable prevalence of NEUROD1‐MODY in the Chinese population is 0.0009%–0.88%.

### 3.3 Summary and Analysis of the Clinical Characteristics of Reported NEUROD1‐MODY Patients

**FIGURE 2:**
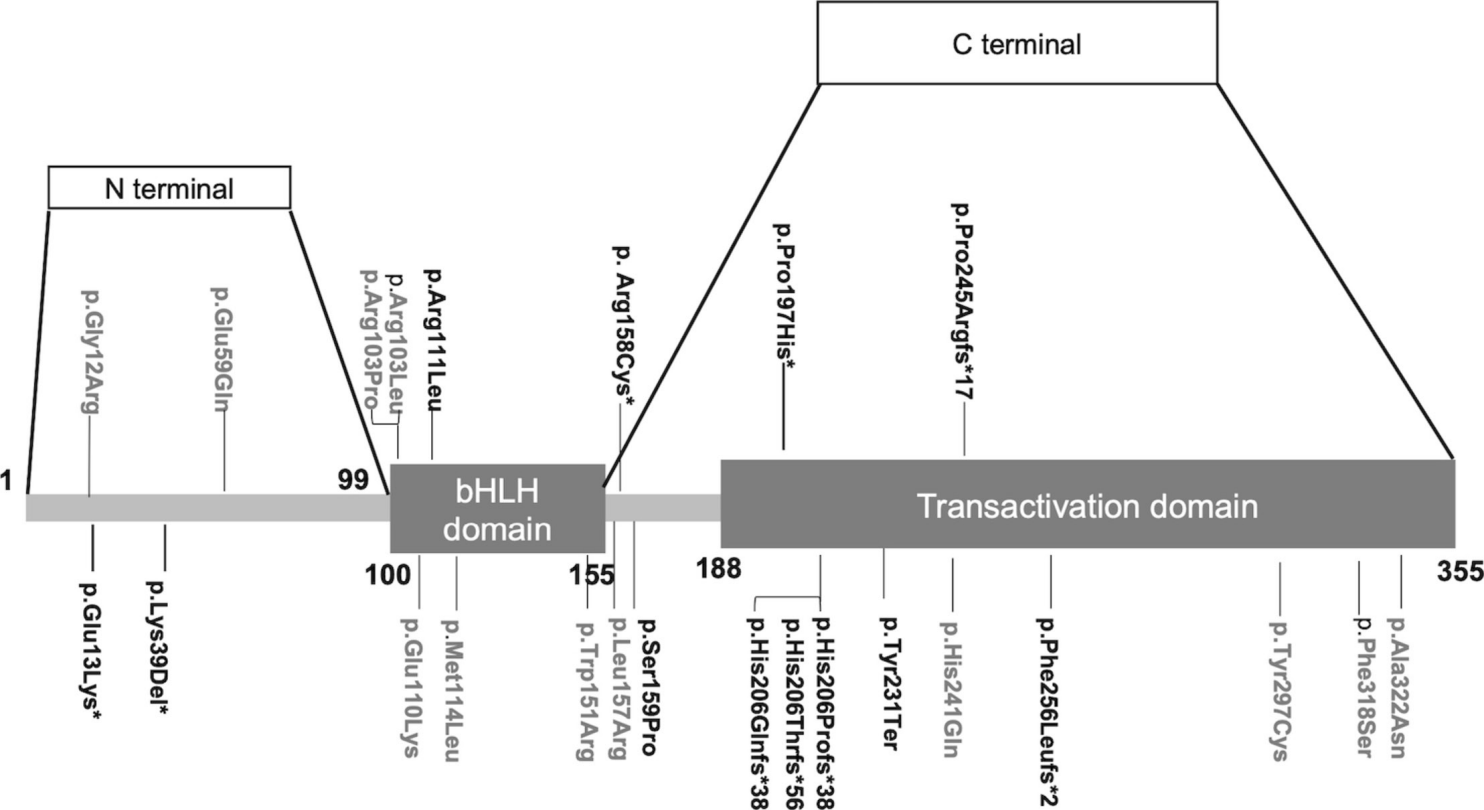
The variants position shown in the NEUROD1 domain. Schematic representation of the NEUROD1 domain structure and the position of reported NEUROD1 variants. Variants described in the EOD cohort were marked with *. Uncertain of significance variants were in gray. Other variants were pathogenic and likely pathogenic variants.

Up to October 30, 2023, 24 papers reporting NEUROD1‐MODY cases were retrieved, including 16 research articles, six case reports, one review, and one letter (Supporting Information: References). Thirty families and 81 patients were enrolled (Table S5). A total of 24 variants of NEUROD1 have been reported. Among them, two were benign and were excluded from the following analysis. The remaining 22 variants, except one mutation c.162G>A which was not located in the coding region, were shown in Figure 2. Only three (13.6%) previously reported mutations were used in in vitro functional studies. Only eight variants (36.4%) were identified as P or LP according to the ACMG guidelines. Others (63.6%) could only be categorized as VUS (Table S3). Thus, 28 (34.6%) patients carrying P or LP variants were diagnosed with NEUROD1‐MODY and enrolled for further analysis. Patients carrying P197H were analyzed separately.

Of the 28 patients, 46.4% were Caucasian and 57.1% were female. The median age at diagnosis was 30 (19, 40) years. A higher proportion of patients were underweight and normal weight (68.2%). Among them, three (10.7%) had a history of ketosis. Most patients had a family history of diabetes (92.9%) and had received a range of therapies including insulin (50%). About 1/3 of the patients had chronic complications. It is worth noting that three patients (10.7%) were complicated with neurological disorders. Among them, two Japanese patients presented with mental retardation and malformations [7], one Italian patient showed a mild speech delay in early childhood [21].

Notably, two Turkish patients carrying the P197H variant who were not included presented neurological disorders with epilepsy and hypopituitarism, either [22]. The proportion of combined neurological abnormalities reached 40% in the five patients with the P197H mutation, including the patient from our EOD cohort. They were all underweight and normal weight (100%). The median age at diagnosis was 12 (5, 19) years. The characteristics of confirmed cases according to the ACMG guidelines and patients with the P197H mutation are summarized in Table 3.

**TABLE 3:** Clinical characteristics of previously reported NEUROD1‐MODY patients.

	*N* = 28 (cases re‐determined by the ACMG guidelines)	*N* = 5 (patients with P197H mutation)
Race		
Asian (*n*%)	11 (39.3%)	1^a^ (20%)
Latin	4 (14.3%)	0
Caucasian	13 (46.4%)	4 (80%)
Sex		
Male	12 (42.9%)	2 (40%)
Female	16 (57.1%)	3 (60%)
Age	30 (19, 40)	12 (5, 19)
BMI (kg/m^2^)	23.72 (19.90, 26.19)	16.2 (15.4, NA)
	***n* = 22**	***n* = 3**
Overweight and obesity (%)	7 (31.8%)	0 (0%)
Underweight and normal (%)	15 (68.2%)	3 (100%)
Ketosis^b^	2 (7.1%)	1 (20%)
Ketosis^c^	3 (10.7%)	1 (20%)
Family history	26 (92.9%)/*n* = 28	1 (33.3)/*n* = 3
HbA1c (%)	8.5 (6.5, 10.5)/*n* = 4	6.3 (5.5, 11.8)
FBG (mmol/L)	7.61 (7.20, 13.89)/*n* = 7	13.4 (5.5, /)/*n* = 3
FCP (ng/ml)	2.40 (1.05, 2.95)/*n* = 5	0.82 (0.39, /)/*n* = 3
Treatment	***n* = 26**	***n* = 5**
Diet	3 (11.5%)	2 (40%)
OAD	10 (38.5%)	1 (20%)
Ins	13 (50.0%)	2 (40%)
Complication	***n* = 7**	***n* = 4**
DPN	0	0
DN	2 (28.6%)	0
DR	2 (28.6%)	0
Neurological abnormalities	3 (10.7%)	2 (40%)
Mutation		
Fs	19 (67.9%)	
Ms	8 (28.6%)	
Ns	1 (3.6%)	
Domain		
bHLH	4 (14.3%)	
Transactivation	20 (71.4%)	

*Note:* Data are medians (interquartile ranges) for skewed variables or numbers (proportions) for categorical variables.

Abbreviations: BMI, body mass index; DN, diabetic nephropathy; DPN, diabetic peripheral neuropathy; DR, diabetic retinopathy; FBG, fasting blood glucose; FCP, fasting c‐peptide; Fs, frameshift mutation; HbA1c, hemoglobin A1c; INS, insulin; LP, likely pathogenic; Ms, missense mutation; NA, not applicable; Ns, nonsense; OAD, oral antidiabetic drugs; P, pathogenic; VUS, uncertain significance.

^a^ The patient from our study cohort.

^b^ Ketosis presenting at diabetes diagnosis.

^c^ Ketosis occurring during the course of diabetes.

We also evaluated the characteristics of the NEUROD1 mutations. Notably, all patients with ketosis or combined neurological malformations had mutations in the transactivation domain (Table S5). Among eight P and LP variants, no Asian patients carried mutations in the bHLH domain, five of six (83.3%) variants located in the transactivation domain were found in Asians and Latins. Therefore, we compared the clinical features of Asian and Caucasian patients (Table 4). Asian patients had lower median BMI (19.95 vs. 25.28 kg/m^2^, respectively, *p* = 0.002) and lower rates of overweight and obesity (0 vs. 66.7%, respectively, *p* = 0.009). In addition, all patients who developed ketosis or experienced the onset of ketosis were Asian.

**TABLE 4:** Compared clinical characteristics between Caucasian and Asian NEUROD1‐MODY patients.

	*N* = 24		*p*
	Caucasian (*n* = 13)	Asian (*n* = 11)	
Sex			
Male	6 (46.2%)	4 (36.4%)	0.697
Female	7 (53.8%)	7 (63.6%)	
Age	36 (18, 40)	31 (14, 61)	0.976
BMI (kg/m^2^)	***n* = 9**	***n* = 9**	
	25.28 (23.74, 29.48)	19.95 (16.58, 22.95)	0.002
Overweight and obesity (%)	6 (66.7%)	0	0.009
Underweight and normal (%)	3 (33.3%)	9 (100%)	
HbA1c (%)	NA	NA	NA
FBG (mmol/L)	NA	7.3 (6.2, 8.2)/*n* = 4	NA
FCP (ng/dl)	NA	2.65 (1.05, 2.98)/*n* = 4	NA
Ketosis^a^	0	3 (27.3%)	
Ketosis^b^	0	2 (18.2%)	
Family history	13 (100%)	9 (81.8%)	0.199
Treatment	***n* = 12**	***n* = 10**	
Diet	1 (8.3%)	1 (10%)	NA
Ins	7 (58.3%)	5 (50%)	
OAD	4 (33.3%)	4 (40%)	
Complication	***n* = 3**	***n* = 4**	
DPN	0	0	NA
DN	0	1 (25%)	
DR	0	1 (25%)	
Neurological abnormalities	1 (7.7%)	2 (18.2)	
Mutation			
Fs	9 (69.2%)	7 (63.6%)	NA
Ms	4 (30.8%)	4 (36.4%)	
Ns	0	0	

*Note:* Data are means ± SD or medians (interquartile ranges) for skewed variables or numbers (proportions) for categorical variables. *p* values are for the *t*‐test, Mann–Whitney *U* test, and *χ*2 analyses, Continuity‐corrected chi‐square test, or Fisher's exact test across the groups. A two‐sided test with *p* < 0 0.05 was considered to be statistically significant.

Abbreviations: BMI, body mass index; DN, diabetic nephropathy; DPN, diabetic peripheral neuropathy; DR, diabetic retinopathy; FBG, fasting blood glucose; FCP, fasting c‐peptide; Fs, frameshift mutation; HbA1c, hemoglobin A1c; INS, insulin; KOD, ketosis onset diabetes; Ms, missense mutation; NA, not applicable; Ns, nonsense mutation; OAD, oral antidiabetic drugs.

^a^ Ketosis presenting at diabetes diagnosis.

^b^ Ketosis occurring during the course of diabetes.

## 4 Discussion

In this study, for the first time we evaluated the prevalence of NEUROD1‐MODY in a representative Chinese sample of EOD. We demonstrated that the P197H mutation of the NEUROD1 gene reduced gene transcription activity to a similar extent as the first reported pathogenic variant (R111L) responsible for NEUROD1‐MODY. These findings indicate that NEUROD1‐MODY is a rare form of monogenic diabetes, with a prevalence of less than or equal to 0.15% (1/679) among Chinese patients with EOD. It is worth noting that racial differences exist. Asian patients are more likely to develop ketosis, have a higher proportion of normal weight or underweight individuals, and mutations often occur in the Transactivation domain. All patients with neurological abnormalities had mutations that occurred in the transactivation domain.

A patient carrying a heterozygous variant of P197H, who was diagnosed with diabetes at 26 years of age, was found in our study. She had a history of diabetes ketosis, low BMI categorized as underweight, and low birth weight. Her mother and second aunt also had diabetes. She was treated with MDI, and without neurological abnormalities. P197H has also been reported in four children with a clinical diagnosis of MODY in Turkey [22, 23]. They were all underweight or normal weight, with onset at a young age (< 25 years‐old), low FCP levels, and various treatments. It is noteworthy that two of them presented neurological disorders including epilepsy and hypopituitarism, which is supposed to be a feature of NEUROD1‐MODY. However, the MAF of P197H differs between Caucasian (0.024) and East Asian (0.00034) populations, leading to a controversial conclusion about pathogenicity according to ACMG guidelines. Nevertheless, from the results of in vitro functional experiments, P197H significantly decreased the transcriptional activity of the insulin promoter, comparable to the well‐known R111L mutation causing NEUROD1‐MODY, which has been identified by Malecki et al. using the same Dual‐Luciferase system, demonstrating the likelihood of pathogenicity in the Chinese population. Therefore, we proposed the hypothesis that P197H may account for MODY in Chinese with a higher penetrance than Caucasian. Most types of monogenic diabetes have reduced penetrance and variable expressivity [24, 25], including HNF1A, HNF4A, and RFX6, which is considered a candidate gene for MODY, emphasizing the role of environment and genetic factors [26, 27]. Studies have demonstrated that a combination of factors such as ethnicity, differential allelic expression, epigenetic modifications, the modulating influence of additional genetic variants, environment, lifestyle, and even age or sex takes into account the differences in penetrance [27]. East Asians have reduced β‐cell function, which may result from a specific genetic background and lead to a high penetrance of P197H [28]. Notably, the Genome‐Wide Association Studies (GWAS) information provides a strong correlation between P197H and random blood glucose level (*p* = 3 × 10^−8^) in up to 459 772 individuals of European ancestry without diabetes, suggesting a probable increasing susceptibility to diabetes in Caucasians despite not causing diabetes directly [29]. A recent study found an association between the homozygote P197H variant and type 1 diabetes mellitus (T1DM) phenotype in a Palestinian family with T1DM [30]. Based on the impairment of NEUROD1 gene function, P197H may worsen the phenotype or increase the penetrance of patients with T1DM. Consequently, it should not be the end of the investigation of P197H. More comprehensive in vitro functional experiments or animal models are needed to focus on P197H in the future.

The precise prevalence of NEUROD1‐MODY remains unclear. Previous large‐scale studies did not include NEUROD1 in their gene panels [9, 10, 11, 31, 32]. Several studies with small sample sizes (*n* = 25–289) [6, 7, 8] have screened NEUROD1 variants in selected patients who were suspected of having monogenic diabetes and showed a relatively high prevalence (1.5%–4%), which factually might have overestimated the prevalence of NEUROD1‐MODY. In our study, the EOD cohort was recruited based only on age at diagnosis, regardless of other clinical features of type 2 diabetes, which is more representative. In vitro functional experiments were performed to evaluate the variants, and stricter diagnostic criteria were established to diagnose NEUROD1‐MODY according to the ACMG guidelines. The prevalence of NEUROD1‐MODY (≤ 0.15%) is as rare as CEL‐MODY (zero patient) [33] and lower than PDX1‐MODY (0.59%) [17] and ABCC8‐MODY (1.5%) [34]. We also proximately estimated the prevalence of NEUROD1‐MODY in the ChinaMAP database as a general population. Only p.S274fs carried by one participant was classified as LP (0.009%) and six participants carried P197H, which is controversial in terms of pathogenicity, suggesting that NEUROD1‐MODY is rare in the Chinese general population, although we do not know whether this individual has diabetes or not.

In our study, four rare variants were detected, only one significantly affected the transcriptional activity of NEUROD1 in in vitro functional experiments. All of them would have been defined as VUS if in vitro functional experiments had not been performed. A recent study reclassified 37 699 VUS variants from 1 689 845 individuals, demonstrating that only about 20% of which were ultimately categorized as P or LP [35]. From our literature review, 24 heterozygous mutations were reported to cause NEUROD1‐MODY. We reevaluated the pathogenicity of these variants and found that only eight mutations (33.3%) were identified as P or LP. Two mutations (8.3%) were classified as benign or likely benign, and more than half of the mutations (58.3%) could only be determined to be VUS. However, only two studies have performed in vitro functional assays to confirm the diagnosis of NEUROD1‐MODY [5, 14]. The result of reevaluation indicated a probable misdiagnosis of previous reports. A published review of NEUROD1‐MODY by Japanese authors also ignored the importance of pathogenicity evaluation [15]. The misdiagnosis obscured the features of NEUROD1‐MODY. Therefore, in vitro functional studies should be performed to assess the pathogenicity to avoid misdiagnosis of NEUROD1‐MODY, better describe the clinical features of NEUROD1‐MODY, and estimate its prevalence.

After reevaluating the diagnosis and summarizing the clinical features of reported NEUROD1‐MODY patients, we found that, overall, they conformed to the characteristics of MODY. A greater proportion of patients were underweight or normal weight (68.2%). Fortunately, neurological abnormalities are uncommon in NEUROD1‐MODY patients. Only three of the previously reported 28 NEUROD1‐MODY patients (10.7%) had neurological abnormalities. Although NEUROD1 also plays an important role in neuronal differentiation [36, 37], all patients with PNDM present neurological abnormalities with homozygous NEUROD1 mutations [38]. However, the clinical phenotypic characteristics of NEUROD1‐MODY were not sufficient to distinguish it from other types of MODY. One possible reason for this is the insufficient description of phenotypic features in the previous literature. For example, as shown in Table 3, only five patients (17.9%) had C‐peptide values and only four (14.3%) had HbA1c values. Furthermore, the lipid metabolism data were lacking in most studies. Another possible reason is that NEUROD1 is upstream in the regulation of pancreatic islet cell functions. Previous studies have shown that it modulates the expression of several MODY genes, including insulin [3], GCK [39], ABCC8 [40], PDX1 [41], and PAX4 [42]. Mutations in NEUROD1 affect multiple downstream genes; therefore, the clinical characteristics become less distinct.

Interestingly, racial differences exist in mutation locations and phenotypes. We found that Asian patients had lower rates of obesity or overweight and were more likely to present with ketosis than Caucasian patients. Poorer medical care and later diagnosis of Asian patients should be taken into account [43]. Furthermore, previous studies revealed that East Asians have reduced β‐cell function and that diabetes developed at a lower degree of insulin resistance, which may partly explain these differences [28]. Another possible explanation for this difference in the clinical features may be genetic differences. The bHLH domain is required for heterodimerization with the ubiquitous HLH protein E47. The Transactivation domain is essential for binding to a critical E‐box motif on the INS promoter and is important for insulin gene transcription. In Asian patients, mutations often occur in the transactivation domain, which may directly affect insulin transcription [3]. Therefore, insulin secretion deficiency may be more serious in Asian patients with NEUROD1‐MODY, which causes ketosis. Additionally, we found that all patients with concomitant neurological diseases carried mutations in the transactivation domain. This mechanism can be explained through basic research. A previous study showed that XNDΔ156‐251, located in the transactivation domain, showed neural cell adhesion molecule (N‐CAM) staining. The N‐terminus was not important in the process of neurogenesis and that bHLH was also insufficient for neurogenesis [3]. Nevertheless, due to the small sample size of the current study and lack of description of neurological phenotype in some cases, the conclusion needs to be further proved.

Our study had several strengths. First, the study population was a relatively large Chinese EOD population based on age at diagnosis only, whereas previous studies had mostly used selected populations like suspicious monogenic diabetes patients. Second, many patients carrying VUS variants were diagnosed with NEUROD1‐MODY in prior research without functional experiments. The functional experiments we did evaluated variants and diagnosed NEUROD1‐MODY more accurately. Last but not least, with a more exact diagnosis of reported cases, our literature review yielded more precise clinical characteristics of NEUROD1‐MODY than the prior review.

There were some limitations to our study. First, due to limitations in DNA sequencing, large fragment deletions could not be identified in this study, which may have underestimated the prevalence of NEUROD1‐MODY. Second, although we verified that the variants reduced the transcriptional activity of the insulin promoter using the Dual‐Luciferase Reporter Gene System, further studies at the protein level or in vivo experiments are lacking. Third, we did not collect blood samples from patients' family members in this study, so we are unable to complete the segregation analysis. Fourth, although the sample size of our study is the largest so far to explore the prevalence of MODY in Chinese patients with EOD, it is relatively small compared to China's population. Fifth, due to the rarity of NEUROD1, the racial differences we found were limited to a small sample size and require further validation.

## 5 Conclusion

The prevalence of NEUROD1‐MODY in Chinese patients with EOD is exceedingly low, less than or equal to 0.15% (≤ 1/679). The P197H variant might result in MODY in Chinese with a higher penetrance than Caucasian and needs further exploration. Moreover, differences in the phenotypes and genetic mutations of NEUROD1‐MODY exist among different ethnic populations. Although NEUROD1 plays an important role in neuronal differentiation, neurological abnormalities are uncommon in NEUROD1‐MODY patients.

## Ethics Statement

This study was approved by the Ethics Committee of Peking University People's Hospital ([2013] No. 12), and written informed consent was obtained from all participants.

## Conflicts of Interest

The authors declare no conflicts of interest.

## Supporting information


Data S1.

## References

[1] M. H. Shepherd , B. M. Shields , M. Hudson , et al., “A UK Nationwide Prospective Study of Treatment Change in MODY: Genetic Subtype and Clinical Characteristics Predict Optimal Glycaemic Control After Discontinuing Insulin and Metformin,” Diabetologia 61, no. 12 (2018): 2520–2527, 10.1007/s00125-018-4728-6. 30229274 PMC6223847

[2] N. A. ElSayed , G. Aleppo , V. R. Aroda , et al., “Addendum. 2. Classification and Diagnosis of Diabetes: Standards of Care in Diabetes—2023,” Diabetes Care 46, no. 1 (2023): S19–S40, 10.2337/dc23-ad08. 37356047 PMC10552401

[3] A. Sharma , M. Moore , E. Marcora , et al., “The NeuroD1/BETA2 Sequences Essential for Insulin Gene Transcription Colocalize With Those Necessary for Neurogenesis and p300/CREB Binding Protein Binding,” Molecular and Cellular Biology 19, no. 1 (1999): 704–713, 10.1128/MCB.19.1.704. 9858593 PMC83927

[4] O. Rubio‐Cabezas , J. A. Minton , I. Kantor , D. Williams , S. Ellard , and A. T. Hattersley , “Homozygous Mutations in NEUROD1 Are Responsible for a Novel Syndrome of Permanent Neonatal Diabetes and Neurological Abnormalities,” Diabetes 59 (2010): 2326–2331, 10.2337/db10-0011. 20573748 PMC2927956

[5] M. T. Malecki , U. S. Jhala , A. Antonellis , et al., “Mutations in NEUROD1 Are Associated With the Development of Type 2 Diabetes Mellitus,” Nature Genetics 23 (1999): 323–328, 10.1038/15500. 10545951

[6] G. D. Abreu , R. M. Tarantino , P. H. Cabello , et al., “The First Case of NEUROD1‐MODY Reported in Latin America,” Molecular Genetics & Genomic Medicine 7 (2019): e989, 10.1002/mgg3.989. 31578821 PMC6900366

[7] Y. Horikawa , M. Enya , H. Mabe , et al., “NEUROD1‐Deficient Diabetes (MODY6): Identification of the First Cases in Japanese and the Clinical Features,” Pediatric Diabetes 19 (2018): 236–242, 10.1111/pedi.12553. 28664602

[8] V. Mohan , V. Radha , T. T. Nguyen , et al., “Comprehensive Genomic Analysis Identifies Pathogenic Variants in Maturity‐Onset Diabetes of the Young (MODY) Patients in South India,” BMC Medical Genetics 19 (2018): 22, 10.1186/s12881-018-0528-6. 29439679 PMC5811965

[9] X. Donath , C. Saint‐Martin , D. Dubois‐Laforgue , et al., “Next‐Generation Sequencing Identifies Monogenic Diabetes in 16% of Patients With Late Adolescence/Adult‐Onset Diabetes Selected on a Clinical Basis: A Cross‐Sectional Analysis,” BMC Medicine 17, no. 1 (2019): 132, 10.1186/s12916-019-1363-0. 31291970 PMC6621990

[10] A. Carlsson , M. Shepherd , S. Ellard , et al., “Absence of Islet Autoantibodies and Modestly Raised Glucose Values at Diabetes Diagnosis Should Lead to Testing for MODY: Lessons From a 5‐Year Pediatric Swedish National Cohort Study,” Diabetes Care 43 (2020): 82–89, 10.2337/dc19-0747. 31704690 PMC6925576

[11] F. Mifsud , C. Saint‐Martin , D. Dubois‐Laforgue , et al., “Monogenic Diabetes in Adults: A Multi‐Ancestry Study Reveals Strong Disparities in Diagnosis Rates and Clinical Presentation,” Diabetes Research and Clinical Practice 188 (2022): 109908, 10.1016/j.diabres.2022.109908. 35533745

[12] L. S. de Santana , L. A. Caetano , A. D. Costa‐Riquetto , et al., “Targeted Sequencing Identifies Novel Variants in Common and Rare MODY Genes,” Molecular Genetics & Genomic Medicine 7 (2019): e962, 10.1002/mgg3.962. 31595705 PMC6900361

[13] S. Richards , N. Aziz , S. Bale , et al., “Standards and Guidelines for the Interpretation of Sequence Variants: A Joint Consensus Recommendation of the American College of Medical Genetics and Genomics and the Association for Molecular Pathology,” Genetics in Medicine 17 (2015): 405–424, 10.1038/gim.2015.30. 25741868 PMC4544753

[14] L. Liu , H. Furuta , A. Minami , et al., “A Novel Mutation, Ser159Pro in the NeuroD1/BETA2 Gene Contributes to the Development of Diabetes in a Chinese Potential MODY Family,” Molecular and Cellular Biochemistry 303 (2007): 115–120, 10.1007/s11010-007-9463-0. 17440689

[15] Y. Horikawa and M. Enya , “Genetic Dissection and Clinical Features of MODY6 (NEUROD1‐MODY),” Current Diabetes Reports 19 (2019): 12, 10.1007/s11892-019-1130-9. 30793219

[16] American Diabetes Association , “9. Pharmacologic Approaches to Glycemic Treatment: Standards of Medical Care in Diabetes—2020,” Diabetes Care 43 (2020): S98–S110, 10.2337/dc20-S009. 31862752

[17] H. Lian , S. Gong , M. Li , et al., “Prevalence and Clinical Characteristics of PDX1 Variant Induced Diabetes in Chinese Early‐Onset Type 2 Diabetes,” Journal of Clinical Endocrinology and Metabolism 108, no. 12 (2023): e1686–e1694, 10.1210/clinem/dgad303. 37279936

[18] C. Chen , F. C. Lu , and Department of Disease Control Ministry of Health PRC , “The Guidelines for Prevention and Control of Overweight and Obesity in Chinese Adults,” Biomedical and Environmental Sciences 17 (2004): 1–36. 15807475

[19] K. C. Tan and Consultation WHOE , “Appropriate Body‐Mass Index for Asian Populations and Its Implications for Policy and Intervention Strategies,” Lancet 363 (2004): 157–163, 10.1016/S0140-6736(03)15268-3. 14726171

[20] World Health Organization , “Obesity: Preventing and Managing the Global Epidemic. Report of a WHO Consultation,” in World Health Organization Technical Report Series, vol. 894 (WHO, 2000), 1–253. 11234459

[21] M. Lezzi , C. Aloi , A. Salina , et al., “Diabetes Mellitus Diagnosed in Childhood and Adolescence With Negative Autoimmunity: Results of Genetic Investigation,” Frontiers in Endocrinology 13 (2022): 894878, 10.3389/fendo.2022.894878. 35769090 PMC9235348

[22] S. Y. Agladioglu , Z. Aycan , S. Cetinkaya , et al., “Maturity Onset Diabetes of Youth (MODY) in Turkish Children: Sequence Analysis of 11 Causative Genes by Next Generation Sequencing,” Journal of Pediatric Endocrinology & Metabolism 29 (2016): 487–496, 10.1515/jpem-2015-0039. 26669242

[23] D. K. Demirci , F. Darendeliler , S. Poyrazoglu , et al., “Monogenic Childhood Diabetes: Dissecting Clinical Heterogeneity by Next‐Generation Sequencing in Maturity‐Onset Diabetes of the Young,” Omics—A Journal of Integrative Biology 25 (2021): 431–449, 10.1089/omi.2021.0081. 34171966

[24] R. Kingdom and C. F. Wright , “Incomplete Penetrance and Variable Expressivity: From Clinical Studies to Population Cohorts,” Frontiers in Genetics 13 (2022): 920390. 35983412 10.3389/fgene.2022.920390 PMC9380816

[25] M. Li , N. Popovic , Y. Wang , C. Chen , and C. Polychronakos , “Incomplete Penetrance and Variable Expressivity in Monogenic Diabetes; a Challenge but Also an Opportunity,” Reviews in Endocrine & Metabolic Disorders 24 (2023): 673–684, 10.1007/s11154-023-09809-1. 37165203

[26] U. L. Mirshahi , K. Colclough , C. F. Wright , et al., “Reduced Penetrance of MODY‐Associated HNF1A/HNF4A Variants but Not GCK Variants in Clinically Unselected Cohorts,” American Journal of Human Genetics 109 (2022): 2018–2028, 10.1016/j.ajhg.2022.09.014. 36257325 PMC9674944

[27] K. A. Patel , J. Kettunen , M. Laakso , et al., “Heterozygous Protein Truncating Variants Are Associated With MODY With Reduced Penetrance,” Nature Communications 8 (2017): 888, 10.1038/s41467-017-00895-9. PMC5638866 29026101

[28] K. Kodama , D. Tojjar , S. Yamada , K. Toda , C. J. Patel , and A. J. Butte , “Ethnic Differences in the Relationship Between Insulin Sensitivity and Insulin Response A Systematic Review and Meta‐Analysis,” Diabetes Care 36 (2013): 1789–1796, 10.2337/dc12-1235. 23704681 PMC3661854

[29] V. Lagou , L. D. Jiang , A. Ulrich , et al., “GWAS of Random Glucose in 476,326 Individuals Provide Insights Into Diabetes Pathophysiology, Complications and Treatment Stratification,” Nature Genetics 55 (2023): 1448–1461, 10.1038/s41588-023-01462-3. 37679419 PMC10484788

[30] A. Bawatneh , A. Darwish , H. Eideh , and H. M. Darwish , “Identification of Gene Mutations Associated With Type 1 Diabetes by Next‐Generation Sequencing in Affected Palestinian Families,” Frontiers in Genetics 14 (2023): 1292073, 10.3389/fgene.2023.1292073. 38274107 PMC10808782

[31] V. Bansal , J. Gassenhuber , T. Phillips , et al., “Spectrum of Mutations in Monogenic Diabetes Genes Identified From High‐Throughput DNA Sequencing of 6888 Individuals,” BMC Medicine 15 (2017): 213, 10.1186/s12916-017-0977-3. 29207974 PMC5717832

[32] M. Harsunen , J. L. T. Kettunen , T. Harkonen , et al., “Identification of Monogenic Variants in More Than Ten per Cent of Children Without Type 1 Diabetes‐Related Autoantibodies at Diagnosis in the Finnish Pediatric Diabetes Register,” Diabetologia 66 (2023): 438–449, 10.1007/s00125-022-05834-y. 36418577 PMC9892083

[33] S. Y. Sun , S. Q. Gong , M. Li , et al., “Clinical and Genetic Characteristics of CEL‐MODY (MODY8): A Literature Review and Screening in Chinese Individuals Diagnosed With Early‐Onset Type 2 Diabetes,” Endocrine 83, no. 1 (2024): 99–109, 10.1007/s12020-023-03512-6. 37726640

[34] M. Li , S. Q. Gong , X. Y. Han , et al., “Genetic Variants of ABCC8 and Phenotypic Features in Chinese Early Onset Diabetes,” Journal of Diabetes 13, no. 7 (2021): 542–553, 10.1111/1753-0407.13144. 33300273

[35] E. L. E. Chen , F. M. Facio , K. W. Aradhya , et al., “Rates and Classification of Variants of Uncertain Significance in Hereditary Disease Genetic Testing,” JAMA Network Open 6 (2023): e2339571, 10.1001/jamanetworkopen.2023.39571. 37878314 PMC10600581

[36] J. H. Cho and M. J. Tsai , “The Role of BETA2/NeuroD1 in the Development of the Nervous System,” Molecular Neurobiology 30 (2004): 35–47, 10.1385/MN:30:1:035. 15247487

[37] E. M. Morrow , T. Furukawa , J. E. Lee , and C. L. Cepko , “NeuroD Regulates Multiple Functions in the Developing Neural Retina in Rodent,” Development 126 (1999): 23–36, 10.1242/dev.126.1.23. 9834183

[38] H. Demirbilek , N. Hatipoglu , U. Gul , et al., “Permanent Neonatal Diabetes Mellitus and Neurological Abnormalities due to a Novel Homozygous Missense Mutation in NEUROD1,” Pediatric Diabetes 19, no. 5 (2018): 898–904, 10.1111/pedi.12669. 29521454

[39] J. M. Moates , S. Nanda , M. A. Cissell , M. J. Tsai , and R. Stein , “BETA2 Activates Transcription From the Upstream Glucokinase Gene Promoter in Islet Beta‐Cells and Gut Endocrine Cells,” Diabetes 52 (2003): 403–408, 10.2337/diabetes.52.2.403. 12540614

[40] J. W. Kim , V. Seghers , J. H. Cho , et al., “Transactivation of the Mouse Sulfonylurea Receptor I Gene by BETA2/NeuroD,” Molecular Endocrinology 16 (2002): 1097–1107, 10.1210/mend.16.5.0934. 11981044

[41] S. Sharma , U. S. Jhala , T. Johnson , K. Ferreri , J. Leonard , and M. Montminy , “Hormonal Regulation of an Islet‐Specific Enhancer in the Pancreatic Homeobox Gene STF‐1,” Molecular and Cellular Biology 17 (1997): 2598–2604, 10.1128/Mcb.17.5.2598. 9111329 PMC232109

[42] R. Bohuslavova , O. Smolik , J. Malfatti , et al., “NEUROD1 Is Required for the Early α and β Endocrine Differentiation in the Pancreas,” International Journal of Molecular Sciences 22 (2021): 6713, 10.3390/ijms22136713. 34201511 PMC8268837

[43] J. C. N. Chan , V. Malik , W. P. Jia , et al., “Diabetes in Asia Epidemiology, Risk Factors, and Pathophysiology,” JAMA: The Journal of the American Medical Association 301 (2009): 2129–2140, 10.1001/jama.2009.726. 19470990

