# Supplementary material for: Prevalence and Clinical Characteristics of NEUROD1‐MODY in Chinese Early‐Onset Type 2 Diabetes Mellitus and a Literature Review
Source: J Diabetes. 2025 Mar 27;17(4):e70080. doi: 10.1111/1753-0407.70080 (PMC11949730; doi:10.1111/1753-0407.70080)
Supplement: Supplementary file 1 — Data S1. [file JDB-17-e70080-s001.docx]

**Prevalence and Clinical Characteristics of NEUROD1-MODY in Chinese Early-Onset Type 2 Diabetes Mellitus and a Literature Review**

Tianhao Ba^*^_1_, Qian Ren^*^_1_, Siqian Gong_1_ , Meng Li_1_, Hong Lian_1_, Xiaoling Cai_1_, Wei Liu_1_, Yingying Luo_1_, Simin Zhang_1_, Rui Zhang_1_, Lingli Zhou_1_, Yu Zhu_1_, Xiuying Zhang_1_, Jing Chen_1_, Jing Wu_1_, Xianghai Zhou_1_, Yufeng Li_2_, Xirui Wang_3_, Fang Wang_4_, Liyong Zhong_4_, Xueyao Han ^†^ _1_, Linong Ji ^†^ _1_

**Corresponding Author:**

Xueyao Han_1_, Email: [xueyaohan@sina.com](mailto:xueyaohan@sina.com);

Linong Ji_1_, Email: [jiln@bjmu.edu.cn](mailto:jiln@bjmu.edu.cn);

1.Department of Endocrinology and Metabolism, Peking University People's Hospital, Peking University Diabetes Center. No. 11, Xizhimen South Street, Beijing, 100044, China

Xueyao Han^†^ and Linong Ji ^†^contributed equally to this work, Linong Ji will handle correspondence at all stages

| **Supplementary Material Contents**  Supplementary references | **Pages**  **3** |
| --- | --- |
| Table S1 Clinical characteristics of patients with early-onset type 2 diabetes | **6** |
| Table S2. Primers sequences used in sanger sequencing and PCR | **8** |
| Table S3. Pathogenicity prediction and minor allele frequencies (MAF) of previously reported NEUROD1 variants and novel variants identified in Chinese early-onset type 2 diabetes patients | **9** |
| Table S4. Rare variations of NEUROD1 contained in the China Metabolic Analytics Project (ChinaMAP) database | **12** |
| Table S5. The clinical and biochemical features of probands with NEUROD1-MODY reported in previous studies | **15** |
| Figure S1. Sanger sequencing results of patients with rare variants of NEUROD1 identified in early-onset type 2 diabetes mellitus cohort | **21** |

**Supplementary References**

We conducted a literature review of currently reported patients with NEUROD1-MODY and summarized and analyzed the clinical and genetic characteristics of NEUROD1-MODY. As of October 30, 2023, 24 papers reporting NEUROD1-MODY cases were retrieved, including sixteen research articles, ^[1-16]^ six case reports, ^[17-22]^ one review ^[23]^ and one letter, ^[24]^ of which four were in Chinese. ^[11, 12, 19, 20]^ Thirty families and eighty-one NEUROD1-MODY patients were enrolled in this study. A total of twenty-four variants of NEUROD1 have been reported. Among them, two(8.3%) were benign ^[1, 19]^ and were not included in the following analysis. However, only eight mutations (36.4%) were identified as pathogenic or likely pathogenic according to the ACMG guidelines.^[2, 8, 10, 16, 24]^ More than half of the mutations (58.3%) could only be determined to be variants of uncertain significance (VUS).^[4-8, 11, 17, 18, 20]^ We compared our study with a published review of NEUROD1-MODY by Japanese authors.^[23]^ In our study, fifteen additional newly reported families were enrolled, and more cases (eighty-one cases) and mutations (twenty-two mutations) have been summarized.^[11, 13-18, 21, 22]^

1. Zhang D, Zhou Z, Li L, Weng J, Huang G, Jing P*, et al.* Islet autoimmunity and genetic mutations in Chinese subjects initially thought to have Type 1B diabetes. Diabetic Medicine 2006;23:67-71. doi: 10.1111/j.1464-5491.2005.01722.x.

2. Liu L, Furuta H, Minami A, Zheng T, Jia W, Nanjo K*, et al.* A novel mutation, Ser159Pro in the NeuroD1/BETA2 gene contributes to the development of diabetes in a Chinese potential MODY family. Mol Cell Biochem 2007;303:115-120. doi: 10.1007/s11010-007-9463-0.

3. Kristinsson SY, Thorolfsdottir ET, Talseth B, Steingrimsson E, Thorsson AV, Helgason T*, et al.* MODY in Iceland is associated with mutations in HNF-1 alpha and a novel mutation in NeuroD1. Diabetologia 2001;44:2098-2103. doi: DOI 10.1007/s001250100016.

4. Plengvidhya N, Boonyasrisawat W, Chongjaroen N, Jungtrakoon P, Sriussadaporn S, Vannaseang S*, et al.* Mutations of maturity-onset diabetes of the young (MODY) genes in Thais with early-onset type 2 diabetes mellitus. Clinical Endocrinology 2009;70:847-853. doi: 10.1111/j.1365-2265.2008.03397.x.

5. Gonsorcikova L, Pruhova S, Cinek O, Ek J, Pelikanova T, Jorgensen T*, et al.* Autosomal inheritance of diabetes in two families characterized by obesity and a novel H241Q mutation in NEUROD1. Pediatric Diabetes 2008;9:367-372. doi: 10.1111/j.1399-5448.2008.00379.x.

6. Chapla A, Mruthyunjaya MD, Asha HS, Varghese D, Varshney M, Vasan SK*, et al.* Maturity onset diabetes of the young in India - a distinctive mutation pattern identified through targeted next-generation sequencing. Clinical Endocrinology 2015;82:533-542. doi: 10.1111/cen.12541.

7. Szopa M, Ludwig-Galezowska AH, Radkowski P, Skupien J, Machlowska J, Klupa T*, et al.* A family with the Arg103Pro mutation in the NEUROD1 gene detected by next-generation sequencing - Clinical characteristics of mutation carriers. Eur J Med Genet 2016;59:75-79. doi: 10.1016/j.ejmg.2016.01.002.

8. Horikawa Y, Enya M, Mabe H, Fukushima K, Takubo N, Ohashi M*, et al.* NEUROD1-deficient diabetes (MODY6): Identification of the first cases in Japanese and the clinical features. Pediatr Diabetes 2018;19:236-242. doi: 10.1111/pedi.12553.

9. Agladioglu SY, Aycan Z, Cetinkaya S, Bas VN, Onder A, Peltek Kendirci HN*, et al.* Maturity onset diabetes of youth (MODY) in Turkish children: sequence analysis of 11 causative genes by next generation sequencing. J Pediatr Endocrinol Metab 2016;29:487-496. doi: 10.1515/jpem-2015-0039.

10. Lezzi M, Aloi C, Salina A, Fragola M, Bassi M, Strati MF*, et al.* Diabetes Mellitus Diagnosed in Childhood and Adolescence With Negative Autoimmunity: Results of Genetic Investigation. Front Endocrinol (Lausanne) 2022;13:894878. doi: 10.3389/fendo.2022.894878.

11. Deng XM WX, Xiao XH, Ping F. A novel neurogenic differentiation 1 gene mutation of maturitu-onset diabetes of the young type 6: clinical features and molecular genetic mechanism. Chin J Diabetes Mellitus 2019;11:53-57. doi: 10.3760/cma.j.issn.1674⁃5809.2019.01.009.

12. Xueyao Han CL, Linong Ji. Contribution of MODY6 gene in the pathogenesis of familial type 2 diabetes in Chinese population. Natl Med J China, 2005;85:2463-2467. doi.13. de Santana LS, Caetano LA, Costa-Riquetto AD, Franco PC, Dotto RP, Reis AF*, et al.* Targeted sequencing identifies novel variants in common and rare MODY genes. Molecular Genetics & Genomic Medicine 2019;7. doi: ARTN e96210.1002/mgg3.962.

14. Demirci DK, Darendeliler F, Poyrazoglu S, Al ADK, Gul N, Tutuncu Y*, et al.* Monogenic Childhood Diabetes: Dissecting Clinical Heterogeneity by Next-Generation Sequencing in Maturity-Onset Diabetes of the Young. Omics-a Journal of Integrative Biology 2021;25:431-449. doi: 10.1089/omi.2021.0081.

15. Doddabelavangala Mruthyunjaya M, Chapla A, Hesarghatta Shyamasunder A, Varghese D, Varshney M, Paul J*, et al.* Comprehensive Maturity Onset Diabetes of the Young (MODY) Gene Screening in Pregnant Women with Diabetes in India. PLoS One 2017;12:e0168656. doi: 10.1371/journal.pone.0168656.

16. Abreu GD, Tarantino RM, Cabello PH, Zembrzuski VM, da Fonseca ACP, Rodacki M*, et al.* The first case of NEUROD1-MODY reported in Latin America. Molecular Genetics & Genomic Medicine 2019;7. doi: ARTN e98910.1002/mgg3.989.

17. Brodosi L, Baracco B, Mantovani V, Pironi L. NEUROD1 mutation in an Italian patient with maturity onset diabetes of the young 6: a case report. BMC Endocr Disord 2021;21:202. doi: 10.1186/s12902-021-00864-w.

18. Bouillet B, Crevisy E, Baillot-Rudoni S, Gallegarine D, Jouan T, Duffourd Y*, et al.* Whole-exome sequencing identifies the first French MODY 6 family with a new mutation in the NEUROD1 gene. Diabetes Metab 2020;46:400-402. doi: 10.1016/j.diabet.2020.03.001.

19. Peng SL YS, Lu XY, Zhang MB. A case report of Maturity-onset diabetes of the young type 6 (MODY6) with Graves’ disease. Medical Science Journal of Central South China 2020;48:554-557. doi: 10.15972/j.cnki.43-1509/r.2020.05.028.

20. Zhao NN DG, Wu W, Yuan JN, Zang L, Chen XF, Fu JF. A case report of Maturity-onset diabetes of the young type 6 (MODY6) caused by a novel mutation. Chinese Journal of Practical Pediatrics 2019;34:438-441. doi: 10.19538/j.ek2019050626.

21. Celik NB, Lafci NG, Savas-Erdeve S, Cetinkaya S. Stress Induced Hyperglycemia in Early Childhood as a Clue for the Diagnosis of NEUROD1-MODY. J Clin Res Pediatr Endocrinol 2022. doi: 10.4274/jcrpe.galenos.2022.2022-6-15.

22. Below N, Morrison D, McGowan R, Jones GC. Diagnostic pitfalls in a young adult with new diabetes. Endocrinol Diabetes Metab Case Rep 2023;2023. doi: 10.1530/EDM-23-0024.

23. Horikawa Y, Enya M. Genetic Dissection and Clinical Features of MODY6 (NEUROD1-MODY). Curr Diab Rep 2019;19:12. doi: 10.1007/s11892-019-1130-9.

24. Malecki MT, Jhala US, Antonellis A, Fields L, Doria A, Orban T*, et al.* Mutations in NEUROD1 are associated with the development of type 2 diabetes mellitus. Nat Genet 1999;23:323-328. doi: 10.1038/15500.

**Table S1. Clinical characteristics of early onset diabetes (EOD) cohort**

|  | N=679 |
| --- | --- |
| Sex  Male  Female | 465 (68.5%)  214 (31.5%) |
| Age | 29±5.40 |
| BMI(kg/m^2^)  Overweight & Obesity(%)  Underweight & Normal(%) | 27.7±4.78  309 (45.8%)  366 (54.2%) |
| Family history | 546 (546/675, 80.1%) |
| HbA1c (%) | 9.00 (7.30, 10.90) |
| Sample, n | 639 |
| FBG (mmol/L) | 8.11 (6.35, 10.69) |
| Sample, n | 653 |
| FCP (ng/mL) | 2.05 (1.41, 2.92) |
| Sample, n | 526 |
| FINS (uU/ml) | 12.06 (7.43, 18.27) |
| Sample, n | 561 |
| Treatment  OAD  INS | 357 (52.6%)  258 (38.0%) |

1. Data were means ± SD or medians (interquartile ranges) for skewed variables or numbers (proportions) for categorical variables.

2. Abbreviations: BMI, body mass index; EOD, early-onset type 2 diabetes; FBG, fasting blood glucose; FCP, fasting c-peptide; HbA1c, hemoglobin A1c; INS, insulin; OAD: oral antidiabetic drugs.

**Table S2. Primers sequences used in sanger sequencing and PCR.**

| **Primers** | **Sequences (5'to3')** |
| --- | --- |
| 1.H-NEUROD1--F | CCATCAAAGGAAGGGCTGGT |
| 2.H-NEUROD1-R | TCCCTTGTTGAATGTAGGAAATCG |
| 3.H-ACTIN-F | CATGTACGTTGCTATCCAGGC |
| 4.H-ACTIN-R | CTCCTTAATGTCACGCACGAT |
| 5.H-INS-Promoter-F | GTGAGGGCTTTGCTCTCCTG |
| 6.H-INS-Promoter-R | TTTCCGGACCATTTCCCTGG |
| 7.H-NEUROD1(PCR)-F | CGCTCAGCATCAATGGCAAC |
| 8.H-NEUROD1(PCR)-R | GATTGATCCGTGGCTTTGGG |

Primers 1-2, forward primer and reverse primer for sanger sequencing.

Primers 3-8, primers for real-time PCR which was performed to verify transfection success.

**Table S3. Pathogenicity prediction and minor allele frequencies (MAF) of previously reported NEUROD1 variants and novel variants identified in Chinese early-onset type 2 diabetes patients**

| No. | Ref* | DNA change | Protein change | 1000G | ExAC | CADD | Mutation taster | PolyPhen-2 | Evidence of pathogenicity | ACMG Classification |
| --- | --- | --- | --- | --- | --- | --- | --- | --- | --- | --- |
| 1 | 6 | c.-162G>A | / | 0.0006 | NA | 16.52 | Deleterious | / | PM2+PP3 | VUS |
| 2 | 12 | c.34G>C | p.Gly12Arg | 0.001 | 0.000124 | 16.86 | Deleterious | Benign | PM2+PP3 | VUS |
| 3† | / | c.37G>A | p.Glu13Lys | NA | 0.00000824 | 24.3 | disease causing | Possibly damaging | PM2 PP3 | VUS |
| 4† | / | c.115_c.117delAAG | p.Lys39del | NA | 0 | NA | NA | NA | PM2 | VUS |
| 5 | 6,11 | c.175 G>C | p.Glu59Gln | 0.00005 | 0.0004 | 22.2 | Deleterious | Benign | PM2+PP3 | VUS |
| 6 | 20 | c.308G >T | p.Arg103Leu | NA | NA | 32 | Deleterious | Probably damaging | PM2+PP1+PP3+PP4 | VUS |
| 7 | 7 | c.308G>C | p.Arg103Pro | NA | NA | 32 | Deleterious | Probably damaging | PM2+PP3 | VUS |
| 8 | 3 | c.328G>A | p.Glu110Lys | NA | NA | 32 | Deleterious | Probably damaging | PM2+PP1+PP3 | VUS |
| 9 | 24 | c.332G>T | p.Arg111Leu | NA | NA | 32 | Deleterious | Probably damaging | PS3+PM2+PP3 | LP |
| 10 | 17, 18 | c.340A>C | p.Met114Leu | NA | NA | 27.3 | Deleterious | Probably damaging | PM2+PP3 | VUS |
| 11 | 20 | c.451T >C | p.Trp151Arg | NA | 0.0000329 | 29.9 | Deleterious | Probably damaging | PM2+PP1+PP3+PP4 | VUS |
| 12 | 8 | c.470T>G | p.Lue157Arg | NA | NA | 30 | Deleterious | Probably damaging | PM2+PP3 | VUS |
| 13† | / | c.472C>T | p.Arg158Cys | NA | NA | 32 | disease causing | Possibly damaging | PM2+ PP3 | VUS |
| 14 | 2 | c.475T>C | p.Ser159Pro | NA | NA | 22.8 | Deleterious | Probably damaging | PS3+PM2+PP3 | LP |
| 15† | 9, 14, / | c.590C > A | p.Pro197His | 0 | 0.0003 | 23 | BENIGN | Possibly damaging | PS3+BS1+PP3 | VUS |
| 16 | 24, 8 | c.616_617insC | p.His206Profs*38 | NA | 0.000657/79 | NA | NA | NA | PVS1+PS3+PM2 | P |
| 17 | 8 | c.616delC | p.His206Thrfs*56 | NA | NA | NA | NA | NA | PVS1+PS1+PM2 | P |
| 18 | 10 | c.617_618insA | p.His206Gln*38 | NA | NA | NA | NA | NA | PVS1+PS1+PM2 | P |
| 19 | 13 | c.693C > G | p.Tyr231Ter | NA | NA | 34 | disease causing | NA | PS3+PM2+PP3 | LP |
| 20 | 21, 6, 5 | c.723C>G | p.His241Gln | 0.0018 | 0.000989 | 23.1 | Deleterious | Probably damaging | PM2+PP3 | VUS |
| 21 | 8 | c.734delC | p.Pro245Argfs*17 | NA | NA | NA | NA | NA | PVS1+PM2 | LP |
| 22 | 16 | c.766_767del | p.Phe256Leufs*2 | NA | NA | NA | NA | NA | PVS1+PM2 | LP |
| 23 | 22 | c.890A>G | p.Tyr297Cys | NA | NA | 23.5 | disease causing | Benign | PM2+PP3 | VUS |
| 24 | 15 | c.953A>G | p.Phe318Ser | NA | NA | 24.6 | disease causing | Benign | PM2+PP3 | VUS |
| 25 | 4 | c.964_965delinsAA | p.Ala322Asn | NA | NA | NA | NA | NA | PM2 | VUS |

*****: The reference numbers were equal to the order of Supplementary References.

†: Evidence from this study. The MAF of these variants were data in the 1000G_Chinese and ExAC_East Asian database.

Abbreviations: LP, Likely pathogenic; P, Pathogenic; VUS, Uncertain significance; 1000G, Frequency in 1000G (http://browser.1000genomes.org ); ExAC, Frequency in ExAC. (http://exac.broadinstitute.org/)

**Table S4. Rare variations of *NEUROD1* contained in the China Metabolic Analytics Project (ChinaMAP) database**

| No. | DNA change | Amino acid  change | Allele frequency | Count | CADD | Mutation taster | Polyphen2 | Evidence of pathogenicity | ACMG |
| --- | --- | --- | --- | --- | --- | --- | --- | --- | --- |
| 1 | c.34G>C | p.Gly12Arg | 0.000944465 | 20/21176 | 16.86 | Deleterious | Benign | PM2+PP3 | VUS |
| 2 | c.77G>C | p.Cys26Ser | 0.00014167 | 3/21176 | 20.4 | Benign | Probably damaging | PM2+PP3 | VUS |
| 3 | c.113A>G | p.Lys38Arg | 4.72E-05 | 1/21176 | 21.8 | Benign | Benign | PM2+PP3 | VUS |
| 4 | c.141C>A | p.Asn47Lys | 0.00014167 | 3/21176 | 11.59 | Benign | Benign | PM2+BP4 | VUS |
| 5 | c.175G>C | p.Glu59Gln | 9.44E-05 | 2/21176 | 22.2 | Deleterious | Benign | PM2+PP3 | VUS |
| 6 | c.208G>A | p.Glu70Lys | 4.72E-05 | 1/21176 | 23.9 | Benign | Benign | PM2+PP3 | VUS |
| 7 | c.228T>A | p.Asp76Glu | 4.72E-05 | 1/21176 | 12.6 | Benign | Benign | PM2+BP4 | VUS |
| 8 | c.231C>G | p.Asp77Glu | 4.72E-05 | 1/21176 | 12.58 | Benign | Benign | PM2+BP4 | VUS |
| 9 | c.250C>T | p.Arg84Cys | 9.44E-05 | 2/21176 | 31 | Deleterious | Probably damaging | PM2+PP3 | VUS |
| 10 | c.284G>A | p.Arg95His | 4.72E-05 | 1/21176 | 32 | NA | Probably damaging | PM2+PP3 | VUS |
| 11 | c.368A>G | p.Asn123Ser | 4.72E-05 | 1/21176 | 21.1 | Benign | Benign | PM2+PP3 | VUS |
| 12 | c.431G>T | p.Arg144Leu | 4.72E-05 | 1/21176 | 32 | Deleterious | Probably damaging | PM2+PP3 | VUS |
| 13 | c.451T>C | p.Trp151Arg | 4.72E-05 | 1/21176 | 29.9 | Deleterious | Probably damaging | PM2+PP3 | VUS |
| 14 | c.590C>A | p.Pro197His | 0.00028334 | 6/21176 | 23 | Benign | Possibly damaging | PS3+PM2+PP3 | LP |
| 15 | c.607A>C | p.Met203Leu | 4.72E-05 | 1/21176 | 18.21 | Benign | Benign | PM2+PP3 | VUS |
| 16 | c.608T>G | p.Met203Arg | 4.72E-05 | 1/21176 | 21.7 | Benign | Benign | PM2+PP3 | VUS |
| 17 | c.616C>T | p.His206Tyr | 4.72E-05 | 1/21176 | 23.8 | Benign | Probably damaging | PM2+PP3 | VUS |
| 18 | c.634G>C | p.Ala212Pro | 0.0000472 | 1/21176 | 19.85 | NA | Benign | PM2+PP3 | VUS |
| 19 | c.649C>T | p.His217Tyr | 0.00014167 | 3/21176 | 23.7 | Benign | Probably damaging | PM2+PP3 | VUS |
| 20 | c.656A>T | p.Tyr219Phe | 0.0000472 | 1/21176 | 18.68 | Benign | Benign | PM2+PP3 | VUS |
| 21 | c.709T>A | p.Ser237Thr | 0.0000472 | 1/21176 | 23.5 | Benign | Possibly damaging | PM2+PP3 | VUS |
| 22 | c.721C>G | p.His241Asp | 0.0000472 | 1/21176 | 27.7 | NA | Probably damaging | PM2+PP3 | VUS |
| 23 | c.724G>A | p.Val242Ile | 0.0000472 | 1/21176 | 22.6 | Deleterious | Benign | PM2+PP3 | VUS |
| 24 | c.724G>C | p.Val242Leu | 0.0000472 | 1/21176 | 19.81 | Deleterious | Benign | PM2+PP3 | VUS |
| 25 | c.724G>T | p.Val242Phe | 0.0000472 | 1/21176 | 24.5 | Benign | Probably damaging | PM2+PP3 | VUS |
| 26 | c.734C>G | p.Pro245Arg | 0.0000472 | 1/21176 | 24.7 | Benign | Probably damaging | PM2+PP3 | VUS |
| 27 | c.755C>G | p.Ala252Gly | 0.0000944 | 2/21176 | 23.5 | Benign | Possibly damaging | PM2+PP3 | VUS |
| 28 | c.755C>T | p.Ala252Val | 0.0000472 | 1/21176 | 23.5 | Benign | Possibly damaging | PM2+PP3 | VUS |
| 29 | c.772G>A | p.Glu258Lys | 0.00028334 | 6/21176 | 24 | Benign | Possibly damaging | PM2+PP3 | VUS |
| 30 | c.797G>C | p.Ser266Thr | 0.0000472 | 1/21176 | 22.9 | Benign | Benign | PM2+PP3 | VUS |
| 31 | c.820dupA | p.Ser274fs | 0.0000472 | 1/21176 | NA | Deleterious | / | PVS1+PM2+PP3 | P |
| 32 | c.845A>G | p.Asn282Ser | 0.0000944 | 2/21176 | 24.9 | Benign | Probably damaging | PM2+PP3 | VUS |
| 33 | c.938G>A | p.Ser313Asn | 0.0000472 | 1/21176 | 24.5 | Benign | Benign | PM2+PP3 | VUS |
| 34 | c.949A>G | p.Ile317Val | 0.0000944 | 2/21176 | 15.14 | Benign | Benign | PM2+PP3 | VUS |
| 35 | c.964G>A | p.Ala322Thr | 0.000330563 | 7/21176 | 20.1 | Benign | Benign | PM2+PP3 | VUS |
| 36 | c.965C>A | p.Ala322Asp | 0.000330563 | 7/21176 | 23.1 | Benign | Benign | PM2+PP3 | VUS |
| 37 | c.989T>C | p.Ile330Thr | 0.00014167 | 3/21176 | 22.1 | Benign | Benign | PM2+PP3 | VUS |
| 38 | c.993C>A | p.Asp331Glu | 0.0000472 | 1/21176 | 18.49 | Benign | Benign | PM2+PP3 | VUS |
| 39 | c.998T>G | p.Ile333Ser | 0.0000472 | 1/21176 | 24.2 | Benign | Benign | PM2+PP3 | VUS |

Abbreviations: LP, Likely pathogenic; P, Pathogenic; VUS, Uncertain significance.

**Table S5. The clinical and biochemical features of probands with NEUROD1-MODY reported in previous studies**

| **No.** | **Ref*** | **DNA**  **change** | **Amino acid**  **change** | **Race** | **Gender** | **Age at diagnosis** | **BMI**  **(kg/m2)** | **FPG**  **(mmol/L)** | **HbA1c**  **(%)** | **FCP**  **(ng/ml)** | **FINS**  **(uU/ml)** | **Current treatment** |
| --- | --- | --- | --- | --- | --- | --- | --- | --- | --- | --- | --- | --- |
| 1 | 6 | c.-162G>A | / | Asian | M | 30 | 27.5 | 9.7 | NA | 0.52 | 18.2 | OAD |
| 2 | 12 | c.34G>C | p.Gly12Arg | Asian | F | NA | 19.8 | 4 | NA | NA | 14.2 | NA |
| 3 | 12 | c.34G>C | p.Gly12Arg | Asian | M | 54 | 24.9 | 5.1 | NA | NA | 8.4 | NA |
| 4 | 12 | c.34G>C | p.Gly12Arg | Asian | F | 49 | 20 | 6.9 | NA | NA | NA | NA |
| 5 | 12 | c.34G>C | p.Gly12Arg | Asian | F | 34 | 21.7 | 11.2 | NA | NA | 7.8 | NA |
| 6 | 11 | c.175G>C | p.Glu59Gln | Asian | F | 36 | 30.00 | NA | NA | NA | NA | NA |
| 7 | 11 | c.175G>C | p.Glu59Gln | Asian | F | 40 | NA | NA | NA | NA | NA | NA |
| 8 | 6 | c.175G>C | p.Glu59Gln | Asian | M | 30 | 19.31 | NA | NA | NA | NA | OAD |
| 9^‡^ | 20 | c.308G>T | p.Arg103Leu | Asian | M | 8 | 15.49 | 7.1 | 10.6 | NA | NA | INS |
| 10 | 7 | c.308G>C | p.Arg103Pro | Caucasian | F | 38 | 31.1 | 4.3 | 6.8 | 1.3 | NA | Diet |
| 11 | 7 | c.308G>C | p.Arg103Pro | Caucasian | M | 44 | 27.3 | 6.22 | 7.4 | 0.6 | NA | INS |
| 12 | 7 | c.308G>C | p.Arg103Pro | Caucasian | F | 40 | 22.9 | 8.52 | 7.4 | 1.3 | NA | INS |
| 13 | 7 | c.308G>C | p.Arg103Pro | Caucasian | F | 26 | 28 | NA | NA | NA | NA | INS |
| 14 | 7 | c.308G>C | p.Arg103Pro | Caucasian | F | 28 | 25.1 | 6.3 | 7.6 | 0.6 | NA | OAD |
| 15 | 7 | c.308G>C | p.Arg103Pro | Caucasian | F | 50 | NA | 8.31 | 6.5 | 1.6 | NA | OAD |
| 16 | 7 | c.308G>C | p.Arg103Pro | Caucasian | F | 23 | 24 | 4.33 | 5.9 | 1.5 | NA | NA |
| 17 | 3 | c.328G>A | p.Glu110Lys | Caucasian | F | 41 | NA | NA | NA | NA | NA | Diet |
| 18 | 3 | c.328G>A | p.Glu110Lys | Caucasian | F | 68 | NA | NA | NA | NA | NA | Diet |
| 19 | 3 | c.328G>A | p.Glu110Lys | Caucasian | F | 12 | NA | NA | NA | NA | NA | INS |
| 20 | 3 | c.328G>A | p.Glu110Lys | Caucasian | F | 17 | NA | NA | NA | NA | NA | INS |
| 21 | 3 | c.328G>A | p.Glu110Lys | Caucasian | F | 13 | NA | NA | NA | NA | NA | INS |
| 22 | 3 | c.328G>A | p.Glu110Lys | Caucasian | M | 37 | NA | NA | NA | NA | NA | OAD |
| 23 | 3 | c.328G>A | p.Glu110Lys | Caucasian | F | 36 | NA | NA | NA | NA | NA | OAD |
| 24 | 3 | c.328G>A | p.Glu110Lys | Caucasian | F | 44 | NA | NA | NA | NA | NA | OAD |
| 25 | 3 | c.328G>A | p.Glu110Lys | Caucasian | F | 27 | NA | NA | NA | NA | NA | OAD |
| 26 | 3 | c.328G>A | p.Glu110Lys | Caucasian | M | 19 | NA | NA | NA | NA | NA | OAD |
| 27 | 3 | c.328G>A | p.Glu110Lys | Caucasian | F | 37 | NA | NA | NA | NA | NA | OAD |
| 28 | 3 | c.328G>A | p.Glu110Lys | Caucasian | F | 25 | NA | NA | NA | NA | NA | OAD |
| 29† | 24 | c.332G>T | p.Arg111Leu | Caucasian | F | 40 | 28.57 | NA | NA | NA | NA | INS |
| 30† | 24 | c.332G>T | p.Arg111Leu | Caucasian | M | 30 | 28.7 | NA | NA | NA | NA | INS |
| 31† | 24 | c.332G>T | p.Arg111Leu | Caucasian | M | 59 | NA | NA | NA | NA | NA | INS |
| 32† | 24 | c.332G>T | p.Arg111Leu | Caucasian | F | 40 | 30.25 | NA | NA | NA | NA | OAD |
| 33 | 17 | c.340A>C | p.Met114Leu | Caucasian | F | 18 | 34 | NA | 9.1 | 2.2 | NA | INS |
| 34 | 17 | c.340A>C | p.Met114Leu | Caucasian | F | 38 | 31.6 | NA | 8.7 | 2.5 | NA | INS |
| 35 | 17 | c.340A>C | p.Met114Leu | Caucasian | M | 16 | 26.8 | NA | 7.3 | 1.8 | NA | INS |
| 36 | 18 | c.340A>C | p.Met114Leu | Caucasian | M | 25 | 21.7 | 6.17 | 8.6 | 0.4 | NA | INS |
| 37 | 20 | c.451T>C | p.Trp151Arg | Asian | F | 5 | NA | NA | 14 | NA | NA | INS |
| 38^‡^ | 8 | c.470T>G | p.Lue157Arg | Asian | F | 10 | 23.9 | 6.9 | 9.1 | NA | NA | INS |
| 39† | 2 | c.475T>C | p.Ser159Pro | Asian | F | 63 | 22.7 | 7.4 | NA | 2.9 | 18.2 | Diet |
| 40† | 2 | c.475T>C | p.Ser159Pro | Asian | M | 50 | 20 | 5.9 | NA | 0.6 | NA | INS |
| 41† | 2 | c.475T>C | p.Ser159Pro | Asian | M | 61 | 19.9 | 7.2 | NA | 2.4 | 8 | OAD |
| 42† | 2 | c.475T>C | p.Ser159Pro | Asian | M | 27 | 23.7 | 8.5 | NA | 3 | 14.7 | OAD |
| 43^c^ | 9 | c.590C>A | p.Pro197His | Caucasian | M | 12.5 | NA | NA | 5.5 | NA | NA | Diet |
| 44^c^ | 9 | c.590C>A | p.Pro197His | Caucasian | M | 12 | NA | NA | 6.3 | NA | NA | Diet |
| 45 | 14 | c.590C>A | p.Pro197His | Caucasian | F | 6 | 19.10 | 5.5 | 5.4 | 2.44 | 7.1 | OAD |
| 46 | 14 | c.590C>A | p.Pro197His | Caucasian | F | 4 | 15.40 | 22.56 | 10.6 | 0.39 | 2.1 | INS |
| 47† | 24 | c.616_617insC | p.His206Profs*38 | Caucasian | F | 38 | 21.85 | NA | NA | NA | NA | Diet |
| 48† | 24 | c.616_617insC | p.His206Profs*38 | Caucasian | F | 33 | 25.06 | NA | NA | NA | NA | INS |
| 49† | 24 | c.616_617insC | p.His206Profs*38 | Caucasian | M | 19 | 25.28 | NA | NA | NA | NA | INS |
| 50† | 24 | c.616_617insC | p.His206Profs*38 | Caucasian | F | 39 | NA | NA | NA | NA | NA | INS |
| 51† | 24 | c.616_617insC | p.His206Profs*38 | Caucasian | M | 56 | 23.74 | NA | NA | NA | NA | OAD |
| 52† | 24 | c.616_617insC | p.His206Profs*38 | Caucasian | F | 18 | 23.74 | NA | NA | NA | NA | OAD |
| 53† | 24 | c.616_617insC | p.His206Profs*38 | Caucasian | M | 17 | 30.52 | NA | NA | NA | NA | OAD |
| 54† | 8 | c.616_617insC | p.His206Profs*38 | Asian | F | 14 | NA | NA | NA | NA | NA | INS |
| 55^†,‡^ | 8 | c.616_617insC | p.His206Profs*38 | Asian | M | 76 | NA | NA | NA | NA | NA | OAD |
| 56† | 8 | c.616_617insC | p.His206Profs*38 | Asian | F | 31 | NA | NA | NA | NA | NA | OAD |
| 57† | 8 | c.616delC | p.His206Thrfs*56 | Asian | F | 12 | 16.2 | NA | 10.9 | NA | NA | INS |
| 58^†,‡^ | 8 | c.616delC | p.His206Thrfs*56 | Asian | F | 27 | 16.7 | NA | NA | NA | NA | INS |
| 59† | 10 | c.617_618insA | p.His206Glnfs*38 | Caucasian | M | 14 | NA | NA | NA | NA | NA | INS |
| 60^†,§^ | 10 | c.617_618insA | p.His206Glnfs*38 | Caucasian | F | NA | NA | NA | NA | NA | NA | NA |
| 61† | 13 | c.693C > G | p.Tyr231Ter | Latin | M | 20 | NA | 7.6 | 6.1 | 1.5 | NA | OAD |
| 62 | 21 | c.723C>G | p.His241Gln | Caucasian | F | 2 | 16.47 | NA | 5.7 | NA | NA | Diet |
| 63 | 5 | c.723C>G | p.His241Gln | Caucasian | F | 19 | 35.4 | NA | NA | NA | NA | Diet |
| 64 | 5 | c.723C>G | p.His241Gln | Caucasian | M | 30 | 36.8 | 5 | NA | NA | NA | INS |
| 65 | 5 | c.723C>G | p.His241Gln | Caucasian | F | 19 | 33.4 | NA | NA | NA | NA | INS |
| 66 | 5 | c.723C>G | p.His241Gln | Caucasian | F | 20 | 34 | 6.1 | NA | NA | NA | INS |
| 67 | 5 | c.723C>G | p.His241Gln | Caucasian | M | 25 | 35.2 | NA | NA | NA | NA | INS |
| 68 | 5 | c.723C>G | p.His241Gln | Caucasian | F | 20 | 36.9 | NA | NA | NA | NA | INS |
| 69 | 5 | c.723C>G | p.His241Gln | Caucasian | M | 51 | 42.2 | NA | NA | NA | NA | OAD |
| 70 | 6 | c.723C>G | p.His241Gln | Asian | M | 28 | 22.8 | NA | NA | NA | NA | OAD |
| 71 | 6 | c.723C>G | p.His241Gln | Asian | F | 24 | 39.7 | NA | NA | NA | NA | OAD |
| 72† | 8 | c.734delC | p.Pro245Argfs*17 | Asian | F | 11 | NA | NA | NA | NA | NA | INS |
| 73^†,‡,§^ | 8 | c.734delC | p.Pro245Argfs*17 | Asian | F | 34 | NA | NA | NA | NA | NA | NA |
| 74^†,§^ | 16 | c.766_767del | p.Phe256Leufs*2 | Latin | F | 23 | 19.9 | 7.61 | 7.5 | NA | NA | INS |
| 75† | 16 | c.766_767del | p.Phe256Leufs*2 | Latin | M | 25 | 25.4 | 18.33 | 9.4 | NA | NA | OAD |
| 76† | 16 | c.766_767del | p.Phe256Leufs*2 | Latin | F | 26 | 18.9 | 13.89 | NA | NA | NA | Diet |
| 77 | 22 | c.890A>G | p.Tyr297Cys | Asian | M | 20 | 28.8 | 10.10 | 10.6 | NA | NA | OAD |
| 78 | 15 | c.953A>G | p.Phe318Ser | Asian | F | 27 | 30.00 | NA | NA | NA | NA | NA |
| 79 | 4 | c.964_965delinsAA | p.Ala322Asn | Asian | F | 14 | 22.23 | 17.76 | 7.6 | NA | NA | NA |
| 80 | 4 | c.964_965delinsAA | p.Ala322Asn | Asian | F | NA | NA | NA | NA | NA | NA | NA |
| 81 | 4 | c.964_965delinsAA | p.Ala322Asn | Asian | F | NA | NA | NA | NA | NA | NA | NA |

*****: The reference numbers were equal to the order of Supplementary References.

†: Patients with pathogenic or likely pathogenic variants that confirmed diagnosed with NEUROD1-MODY.

^‡^: Patients with ketosis.

^§^: Patients with logical abnormalities

Abbreviations: BMI, body mass index; FBG, fasting blood glucose; FINS, fasting serum insulin; FCP, fasting c-peptide; F, Female; HbA1c, hemoglobin A1c; M, Male; NA, Not Applicable; OAD, Oral antidiabetic drugs.


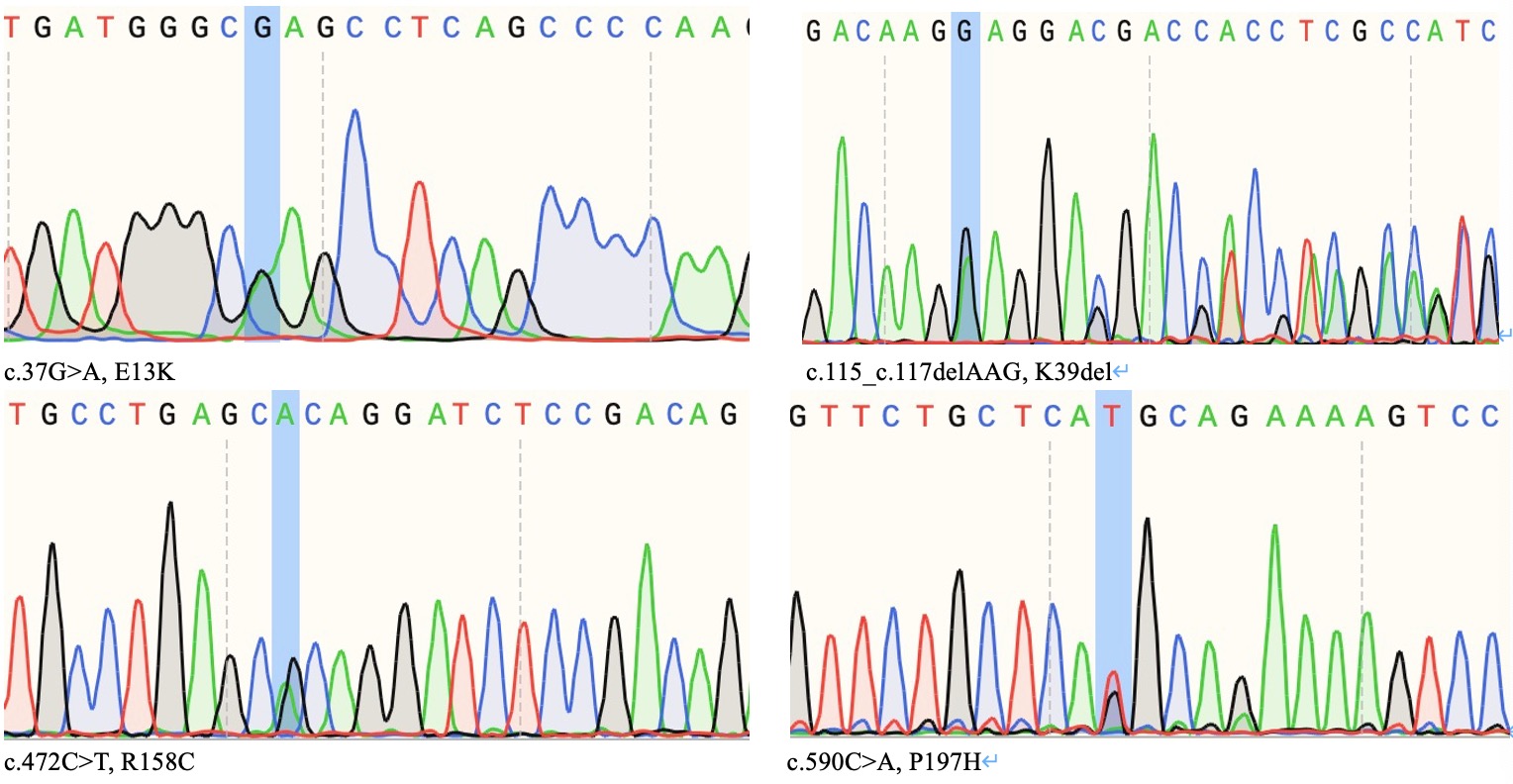


**Figure S1. Sanger sequencing results of patients with rare variants of *NEUROD1* identified in early-onset type 2 diabetes mellitus cohort.**
